# The Multifaceted Uses and Therapeutic Advantages of Nanoparticles for Atherosclerosis Research

**DOI:** 10.3390/ma11050754

**Published:** 2018-05-08

**Authors:** Nicholas DiStasio, Stephanie Lehoux, Ali Khademhosseini, Maryam Tabrizian

**Affiliations:** 1 Biomedical Engineering Department, McGill University, 3775 Rue University, Montréal, QC H3A 2B4, Canada; Nicholas.Distasio@mail.mcgill.ca; 2 Lady Davis Institute for Medical Research, Department of Medicine, McGill University, 3755 Cote Sainte Catherine, Montreal, QC H3T 1E2, Canada; Stephanie.Lehoux@mcgill.ca; 3 Department of Bioengineering, Chemical Engineering, and Radiological Sciences, University of California, Los Angeles, 410 Westwood Plaza, Los Angeles, CA 90095-1600, USA; Khademh@ucla.edu; 4 Faculty of Dentistry, McGill University, 2001 Avenue McGill College #500, Montreal, QC H3A 1G1, Canada

**Keywords:** atherosclerosis, nanoparticles, therapeutic delivery, drug delivery, imaging, targeting

## Abstract

Nanoparticles are uniquely suited for the study and development of potential therapies against atherosclerosis by virtue of their size, fine-tunable properties, and ability to incorporate therapies and/or imaging modalities. Furthermore, nanoparticles can be specifically targeted to the atherosclerotic plaque, evading off-target effects and/or associated cytotoxicity. There has been a wealth of knowledge available concerning the use of nanotechnologies in cardiovascular disease and atherosclerosis, in particular in animal models, but with a major focus on imaging agents. In fact, roughly 60% of articles from an initial search for this review included examples of imaging applications of nanoparticles. Thus, this review focuses on experimental therapy interventions applied to and observed in animal models. Particular emphasis is placed on how nanoparticle materials and properties allow researchers to learn a great deal about atherosclerosis. The objective of this review was to provide an update for nanoparticle use in imaging and drug delivery studies and to illustrate how nanoparticles can be used for sensing and modelling, for studying fundamental biological mechanisms, and for the delivery of biotherapeutics such as proteins, peptides, nucleic acids, and even cells all with the goal of attenuating atherosclerosis. Furthermore, the various atherosclerosis processes targeted mainly for imaging studies have been summarized in the hopes of inspiring new and exciting targeted therapeutic and/or imaging strategies.

## 1. Introduction

Cardiovascular disease is responsible for the deaths of more than 17 million people worldwide and this rate is expected to grow to over 23 million by the year 2030 [1]. More strikingly, it is estimated that there is a death every 40 s attributable to cardiovascular disease [2]. Atherosclerosis is the root cause of the majority of cardiovascular clinical manifestations, which have been significantly curbed due to breakthroughs in drug therapy. However, advanced tools, increasing interest in nanotechnology, and further understanding of the pathology of atherosclerosis elucidated by various animal studies have recently placed researchers in prime position to tackle the complex manifestations of atherosclerosis on a more specific and molecular level. 

Atherosclerosis has typically been viewed as a dietary and lipid accumulation disorder. While lipids certainly play a role in lesion formation, they cannot account for all the concerns of atherosclerosis. It is now known that atherosclerosis begins with endothelial damage that can arise as early as adolescence. Lipids such as apolipoprotein B have special affinity for the basal membrane revealed in areas of damaged endothelium [3]. These regions are well conserved in humans and various animal models and typically occur in curved or branched arteries, which experience disturbed or oscillatory flow dynamics and low shear stress [4].

Endothelial damage permits the accumulation and retention of lipids within the subendothelial space where they can be oxidized by oxidative stress-induced molecules and enzymatic products [5]. Oxidized lipids act as a danger signal to the endothelial cells lining the vessel and the cells begin to increase expression of the inflammatory cell recruitment receptors vascular cell adhesion molecule 1 (VCAM-1), intercellular adhesion molecule 1 (ICAM-1), P-selectin, and E-selectin through inflammatory signaling pathways, such as nuclear factor-κB (NF-κB) [6,7]. These receptors are used as binding moieties by circulating immune cells such as monocytes, which express conjugate ligands such as very late antigen 4 (VLA-4) for VCAM-1 and lymphocyte function-associated antigen 1 (LFA-1) for ICAM-1 [8,9]. The expression of these inflammatory cell recruitment receptors and the production of chemoattractant chemokines lead to increased infiltration of circulating monocytes, which will also express cytokines, thus perpetuating a positive feedback loop.

Upon entering the plaque, monocytes differentiate into macrophages and become activated under the influences of macrophage colony stimulating factor (M-CSF) and tumor necrosis factor-α (TNF-α), both of which are upregulated in plaque cells [10]. These activated macrophages ingest large amounts of lipids via upregulation of scavenger receptors [11], eventually becoming foam cells [12]. These foam cells are prone to apoptosis, releasing damaging cytokines and enzymes that exacerbate the immune response, recruiting more inflammatory cells that amplify the process of plaque formation.

Defective efferocytosis of lipid-laden apoptotic cells also aggravates the situation. Eventually, the formation of a necrotic core, often characterized by the accumulation of cholesterol crystals, marks the transition to a vulnerable plaque that is prone to rupture. In addition to this molecular based degradation, physical destabilization and degradation of the fibrous cap is a consequence of pro-inflammatory signaling. The fibrous cap is composed mostly of collagen secreted by vascular smooth muscle cells migrating into the plaque from the medial layer of the vessel [13]. The upregulation of matrix metalloproteinases (MMP-3 and -9) leads to the degradation of the collagenous cap [10]. These cumulative events are all a result of chronic and non-resolving inflammation [14] that can be countered by anti-inflammatory cytokines, such as interleukin-10 (IL-10) [15]. IL-10 and other anti-inflammatory cytokines help to influence the ratio of activated pro-inflammatory “M1” macrophages to pro-healing “M2” macrophages via the janus kinase/signal transducers and activators of transcription (JAK-STAT) signaling pathway [16]. In the event that the thinned fibrous cap ruptures, damaging lipids, enzymes, cytokines, calcium, and dead cell fragments are released into the blood, stimulating the formation of a thrombus that can quickly occlude the artery, leading to acute clinical events [17,18].

Interest in the potential to apply nanotechnology to cardiovascular disease has been high for some years [19], allowing for developments using nanoparticle research specifically for atherosclerosis. Nanoparticles (NPs) are uniquely suited to combat atherosclerosis given their ability to encapsulate various therapeutics such as nucleic acids, drugs, proteins, and even cells. Encapsulation serves two purposes in the field of nanomedicine: firstly, it protects the in vivo environment from harmful drugs or off-target effects by ensuring that drug release is controlled via material properties and/or targeted to the affected area via surface properties. Secondly, encapsulation protects labile cargo from degradation and/or other unwanted modifications. In addition, the high surface area to volume ratio of nanoparticles makes them ideal for surface functionalization for the purpose of targeting plaque components and/or evading the body’s immune system and clearance. The two most popular moieties incorporated onto nanoparticles are targeting ligands (antibodies, peptides, aptamers, or small molecules) specific for plaque components and PEGylation, which confers stealth and stability in vivo.

**Figure 1:**
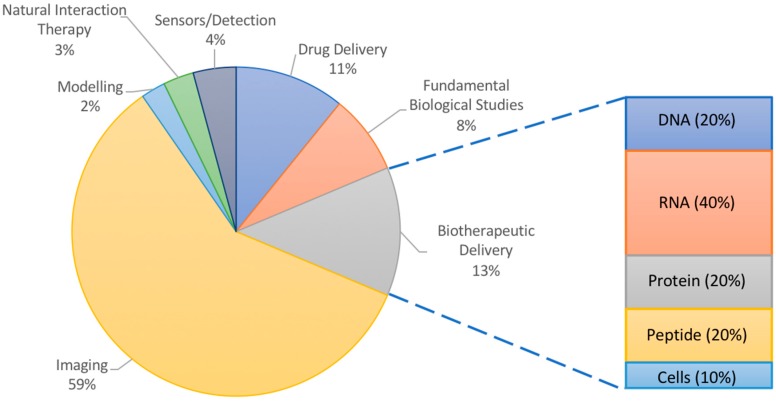
Overall analysis of literature examples chosen for this review. Inset shows percentages of delivered therapeutic in studies for burgeoning field of biotherapeutic delivery with high potential in atherosclerosis research.

Many of the applications of NPs in atherosclerosis have focused on imaging (Figure 1). Ultrasound imaging using contrast-enhancing agents such as gas-filled particles or microbubbles has been ongoing for decades [20]. Though not quite using nanoparticles, ultrasound imaging has nevertheless inspired numerous nano-scale imaging and treatment options, and has been used for atherosclerosis [21,22]. However, there have also been interesting less conventional uses of nanoparticles in modelling, sensing, and elucidating the biology of plaque progression in atherosclerotic mice and hyperlipidemic rabbits. NPs may even participate in the treatment of atherosclerosis, acting by virtue of their material-based natural interactions with plaque components rather than by the delivery of therapeutics. Nanoparticles vary in their molecular makeup, with inorganic components being more useful for studies involving physical phenomena (imaging, photodynamic therapy, etc.) and organic materials being typically chosen for their ability to interact with cargo (protection, encapsulation, controlled release, etc.) and for their increased biocompatibility and biodegradability. However, NPs made of both materials can be targeted to the plaque by fine tuning their size, shape, surface properties, and ligand coating. Exploring these various phenomena can add exciting perspective to the numerous reviews discussing experimental nanoparticle interventions for atherosclerosis, which have focused on imaging or drug delivery [23,24,25]. Thus, this review article illustrates how nanoparticles are advancing the research of atherosclerosis using animal models through the probing of fundamental biological interactions, delivering therapeutics, and reporting back to researchers via imaging or sensing modalities as a function of the materials chosen.

## 2. Influence of Material Properties on NPs Used for Atherosclerosis

In general, the application will guide the choice of material to use for nanoparticle-mediated treatment with two types of interventions relative to atherosclerosis: (1) the delivery of therapeutics and (2) the visualization of plaque and its processes.

### 2.1. Materials Used for Fabrication of Nanoparticles

#### 2.1.1. Polymers

**Figure 2:**
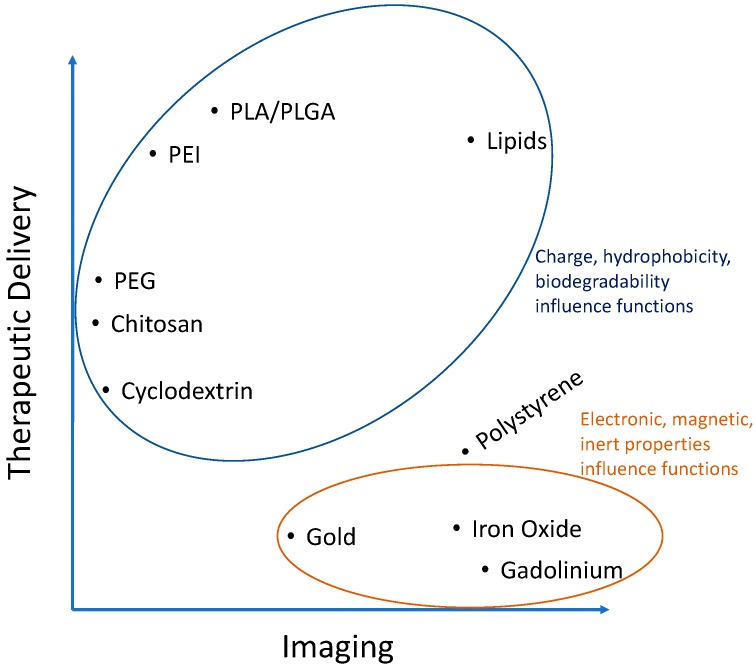
Analysis of Table 1 showing importance of the characteristics of certain materials used to fabricate nanoparticles for atherosclerosis research.

Polymers make up one of the most common groups of materials used to fabricate nanoparticles. This is in part because of their diverse range of fine-tunable properties allowing researchers to control their hydrophobicity, charge, degradability, and many more features. Accordingly, their use in fabricating nanoparticles for atherosclerosis research has experienced an exponential increase and they seem to be the material of choice for therapeutic delivery applications (Table 1). The main attractions for polymers are their typically lower toxicity than metals and the availability of chemically active sites for the functionalization of fluorescent dyes and targeting moieties. They also have an increased carrying capacity for cargo (drugs, proteins, nucleic acids, etc.) related to their biodegradable, polar, charged, and somewhat hydrophobic properties responsible for polymer interaction with, protection of, and controlled release of therapeutics (Figure 2). Some common examples include poly(lactic-co-glycolic acid) (PLGA), polyethylenimine (PEI), poly(L-lysine) (PLL), poly(lactic acid) (PLA), poly(aspartic acid) (PAA), chitosan, gelatin, alginate, and many others and are particularly useful to complex with, protect, and deliver charged polar cargo such as proteins and DNA [26]. A polymer of the monomers lactic and glycolic acid, PLGA is widely used and approved by the U.S. Food and Drug Administration (FDA) and European Medicine Agency (EMA) for some applications [27]. PLGA is biodegradable via hydrolysis of the ester bonds linking monomers and its rate of degradation can be tuned by the percentages of the two monomers. This naturally lends itself to the controlled delivery of therapeutics, an advantageous strategy capable of recapitulating the complex spatio-temporal patterns of signaling within the in vivo plaque environment.

There are also polymers whose function depends on their hydrophobicity and charge but are not biodegradable. These underline the complex array of interactions possible between a nanocarrier and its delivery cargo or imaging modality. This example is best illustrated with polymers formed via stable covalent bonds such as the amide bonds in polyamidoamine (PAMAM) and polyethylenimine (PEI) nanoparticles. These polymers are highly cationic and the long polymer chains render them slightly hydrophobic as well. Their electrostatic binding to anionic plasmid DNA for example is efficient to protect it from degradation, yet it is also desirable to release this DNA once inside the cells so that it can be transcribed into protein. This is why cationic polymers with more biodegradable ester bonds can have increased transfection efficiencies. Though non-viral gene delivery has yet to break into the world of atherosclerosis, biodegradable polymers have high potential [28,29]. However, biodegradability is less important to consider for therapeutics that only have to enter the cell cytoplasm to act such as for RNA interference (RNAi) and even less important for those therapies that can be released outside the cells, such as drugs acting on receptors or being internalized on their own. Thus, the application is the most important factor to consider in choosing a material for NPs. However, the fine-tunability of polymers make them adaptable for many functions within that space and will be discussed more in upcoming sections. For a more complete review of polymers within the context of atherosclerosis, the reader is referred to Lewis et al. [30].

#### 2.1.2. Lipids

Lipid nanoparticles have been around for decades and were some of the first examples of drug delivery tools used by researchers. Typically made from amphiphilic materials, liposome-forming lipids have a long hydrophobic tail of 10–20 carbon atoms sometimes with unsaturated bonds. Emulsification of these lipids with hydrophobic drugs encapsulates them into self-assembled spheres with the hydrophobic lipid tail in the core and typically charged hydrophilic polar head residing in the outer aqueous environment. Thus, the charge and hydrophobic nature of lipids is important for NP formation (Figure 2). Included in the emulsion are often ‘spacer lipids’ such as cholesterol, as in cell membranes. These spheres can be unilamellar or multilamellar depending on post-formation processing techniques. Although most often used to encapsulate hydrophobic imaging agents, drugs, and proteins, there are examples of liposomes for gene delivery, typically by absorbing nucleic acids onto the charged exterior [31]. Furthermore, there is a wide variety of lipids available with already functionalized head groups for the covalent linking of targeting peptides and stealth groups such as polyethylene glycol (PEG). The ratios of all these components are important and must be screened as they often depend on cell type. For example, a targeting ligand density of 1–2.5 mol % of the total lipids used to form particles was found optimal to target cancer cells in vitro and in vivo in a mouse model of human gastric cancer [32].

Liposomes have found particular success for use in atherosclerosis imaging and therapeutic delivery because of their lipid-like properties. The accumulation of low density lipoprotein (LDL) is an initiating event for atherosclerosis inflammation. However, high density lipoprotein (HDL) is athero-protective through an unknown mechanism of transporting lipids and cholesterol from foamy macrophages into the liver for processing [33]. Liposomes have been used to mimic the structure and function of HDL [34,35].

### 2.2. Nanoparticles for Investigating Atherosclerosis

#### 2.2.1. Polystyrene

Polystyrene (PS) is a model biomaterial in terms of its widespread use. It is biocompatible as it is the culture substrate of choice for a wide array of cells. This use has translated to its selection as a nanoparticle material that is stable, relatively inert, and cheap to work with. It can be used to optimize many fine-tunable parameters such as the coating density of targeting ligands onto nanoparticles, helping to isolate the effect of the coating independent of material effects. PS nanoparticles are often commercially made with monodisperse diameters and are available with the chemical functional group of choice already present for linking targeting and imaging moieties. However, even the relatively hydrophobic nature of PS allows for direct adsorption of proteins without the need for chemical functionalization. For example, Pacheco et al. adsorbed specific amounts of Fc receptor ligands onto PS spheres ranging in size from 0.5–2 μm to study the effects of NP size and ligand density on uptake by macrophages [79].

Ligand-coated PS NPs are also internalized by endothelial cells, a process depending in part on the diameter of nanoparticles and density of surface ligands [80]. Chacko et al. described how functionalizing polystyrene NPs with a diameter around 180 nm with antibodies specific for different domains of the platelet endothelial cell adhesion molecule receptor PECAM-1 (CD31) enhances NP uptake in vitro in human umbilical vein endothelial cells (HUVECs) and in vivo in female C57BL/6 mice [81]. They found that binding of a first antibody, added in solution, induces a conformational change in PECAM-1 and that this change unveils a high-affinity domain for the second antibody, coated on NPs, increasing uptake. This process is highly sequence specific as binding of the two antibodies in the reverse order does not show similar success. Nevertheless, the strategy of functionalizing multivalent ligands to NPs typically synergistically enhances their binding to and uptake by endothelial cells, even when targeting different receptors with the same NP [22,63,69]. Thus, polystyrene NPs can be made with discrete diameters and functionalized either physically (adsorption) or chemically (cross-linking) with targeting ligands and fluorescent moieties. However, they offer little in terms of cargo carrying capacity (Figure 2), an important application of nanoparticles. In addition, PS NPs are not used in the clinic due to their inability to biodegrade within the body among other reasons. Still, they are a useful tool to survey in vitro and experimental in vivo binding dynamics and biodistribution [82].

#### 2.2.2. Metallic and Inorganic Materials

One of, if not the most widely used applications of metallic and inorganic material nanoparticles for atherosclerosis is for imaging. Magnetic resonance imaging (MRI) is the classical modality due to the wide availability of both MRI machines and magnetic nanoparticles, typically ultrasmall superparamagnetic nanoparticles of iron oxide (USPIOs) or gadolinium (Gd)-based materials. Studies involving positron emission tomography (PET), computer tomography (CT), ultrasound, and fluorescent/infrared imaging are becoming more common, sometimes in combination with MRI, with potential applications dictated by the choice of NP material.

An advantage of inorganic NPs for imaging is their often smaller size (tens of nm) relative to organic-based NPs, allowing for increased passive uptake. Such a strategy was utilized by Palekar et al. in trying to establish a correlation between the uptake of perfluorocarbon (PFC) NPs as a result of the damaged endothelium and the risk of thrombosis [54]. By incorporating this PFC, the authors were able to do MRI imaging in both the hydrogen and fluorine channels, leading to increased perspectives for detection. Furthermore 3D 2-photon microscopy on en face aortic segments revealed the depth of penetration of the NPs into the plaques of ApoE^−/−^ mice. The accumulation of nano-sized particles via the enhanced permeabilization and retention (EPR) effect is often cited in NP use for various cancers and could be the reason non-targeted imaging NPs have had success already in atherosclerosis. However, researchers are moving toward more specific targeting of plaque components. One recent example of this is an in-depth study by Qiao et al., who used up-converting gadolinium NPs targeted to the plaques of ApoE^−/−^ mice [83]. Coated with an osteopontin antibody, these MRI-detectable NPs localize to macrophages within the plaque. The specific targeting of plaque macrophages has been difficult because they are not very different than macrophages present throughout the body. However, the authors identified osteopontin as a secreted marker of macrophages, particularly foamy cells within the plaque. The nanoformulation was able to resolve small distances live in vivo via up-conversion luminescence imaging in an arterial cuff model capable of recapitulating low and oscillatory shear stresses in the vessel.

Interest is on the rise in targeting the plaque specifically. Many studies have identified possible targets that are overexpressed or disproportionately expressed at the plaque. In addition, advances in chemistry, nanotechnology, and biological knowledge have allowed for the development of specific probes to illuminate some of the processes that drive plaque progression. These are summarized in Table 2.

### 2.3. Therapeutic Strategies Reliant on Nanoparticle Material Properties

**Figure 3:**
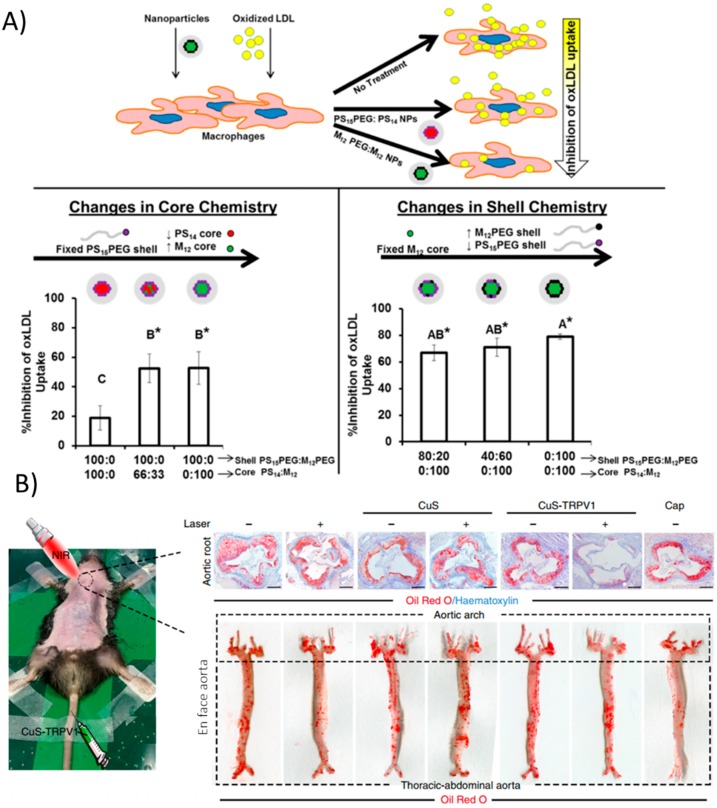
Nanoparticle strategies arising from material properties. (**A**) Core-shell formulations of PEG, polystyrene, and mucic acid of varying compositions modulate physical properties. Increasing hydrophobic cores mimic modified LDL and can compete with its uptake via scavenger receptors, ultimately reducing macrophage lipid uptake. (*) indicates statistical significance from oxLDL control (p ≤ 0.05). Reproduced (adapted) with permission from [105]; (B) CuS nanoparticles act as infrared thermotransducers to control cationic channels important for atherosclerosis processes. Upon irradiation, CuS NPs targeted to TRPV1 heat up and trigger the cation channel to open, allowing a flood of calcium and activation of autophagy processes, preventing atherosclerosis in the aortic root as well as in the entire aorta. Reproduced (adapted) from [108].

There are a few interesting and recent examples of nanoparticles that exert their therapeutic potential by virtue of just their material properties (i.e., there is no delivered therapeutic). Many studies have devised strategies to interfere with the uptake of low density lipoprotein (LDL) by macrophages. As described above, this is a key event in the progression of atherosclerosis. Macrophages take up oxidized LDL via scavenger receptor-A (SR-A) and scavenger receptor B (CD36). Petersen et al. developed amphiphilic nanoparticles with hydrophobic cores composed of mucic acid and polystyrene as well as amphiphilic core-shell NPs consisting of mucic acid-functionalized PEG and polystyrene in vitro in human monocyte-derived macrophages [105]. The lipid-like hydrophobic core-containing NPs act at two different levels to prevent the uptake of lipids. First, they directly compete with the uptake of oxidized LDL via scavenger receptors SR-A and CD36. Secondly, NP uptake led to a decrease in the surface and gene expression of one or both of these receptors for 48h after incubation with the nanoparticles in vitro (Figure 3A). Though the authors did not check how this impacted the formation of foam cells specifically, the processes diminished by NPs (LDL uptake and scavenger receptor expression) are what give rise to foam cells in vivo. The authors further pointed out that the result is similar to the effects of α-tocopherol, though the pathways for this molecule are not well known. Still these are relatively safe materials (although polystyrene could be substituted for a more biodegradable polymer in the future) and represent an interesting strategy to deal with a major issue in atherosclerosis. Other attempts for this strategy have been documented as well [106,107]. However, longer term studies should be performed with this strategy and in vivo to ensure that NP uptake is not mimicking the effects of LDL uptake, just substituting one molecule for another.

Lipid transport primarily occurs in macrophages and other professional phagocytic cells, however, vascular smooth muscle cells (VSMCs) also contribute significantly to lipid accumulation in plaques [13]. A recent study actually targeted VSMCs with copper sulfate (CuS) nanoparticles that acted as a photothermal switch inducing autophagy [108]. Coating the NPs with an antibody against TRPV1, a cation channel, allowed the CuS NPs to specifically accumulate in VSMCs expressing TRPV1 in vitro. Upon irradiation with NIR light, a local increase in temperature was seen due to the NP materials. The heat-activated channel allowed an influx of calcium ions, which activates autophagy, a mechanism by which modified LDL is converted back into free cholesterol for efflux from the cell. Injection of the NPs in vivo in ApoE^−/−^ mice followed by irradiation greatly reduced plaque formation as evidenced by Oil Red O staining (Figure 3B). This simple yet highly effective strategy demonstrated precise in vivo targeting and control of a cellular process that is important in the progression of atherosclerosis, namely the processing of lipids.

Foam cells of macrophage or VSMC origin are characterized by the ingestion of lipids that render them vulnerable to apoptosis and necrosis. In conditions of defective efferocytosis, excessive extracellular cholesterol may lead to the formation of crystal structures, which can activate complex inflammation pathways [14]. In a recent interesting approach to attenuate inflammation, Zimmer et al. delivered cyclodextrin to the plaques of ApoE^−/−^ mice in an attempt to increase cholesterol solubility and removal from the plaque through classical pathways [109]. Though this strategy involved free cyclodextrin oligosaccharides, it could be reimagined to encapsulate cyclodextrin as a therapeutic within a nanoparticle or use it as a component to fabricate the nanoparticle itself as cyclodextrin is widely used to formulate NPs [56,110].

## 3. Nanoparticle-Mediated Delivery of Therapeutics in Atherosclerosis

### 3.1. Delivery of Biotherapeutics

**Figure 4:**
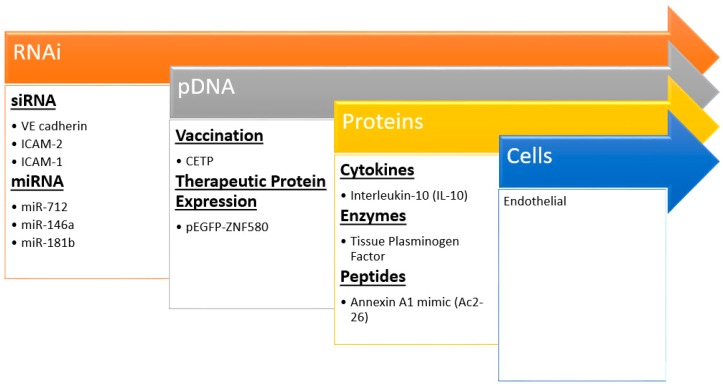
Recent examples of nanoparticle biotherapeutics and their targets in preventing atherosclerosis. Insights and techniques gained from siRNA studies has led to the delivery of larger and more complex molecules even up to cells.

Nanocarriers are increasingly being incorporated into imaging and drug delivery in cardiovascular disease [111,112]. Moreover, there is a burgeoning area of therapeutic delivery including nucleic acids, proteins, and more recently cells (Figure 4) that could be deemed biotherapeutic delivery. Nanoparticles can bring the promises of non-invasive and safe gene therapy, cellular repopulation, and protein delivery to fruition for atherosclerosis. Many significant challenges remain to delivering these biotherapeutics; each cargo comes with its own unique set of design criteria and constraints. However, these strategies hold the most promise for novel effective treatments as evidenced by extensive plaque regression and reduction in typical inflammatory markers after treatment in animal models (Table 3).

#### 3.1.1. RNA Delivery

**Figure 5:**
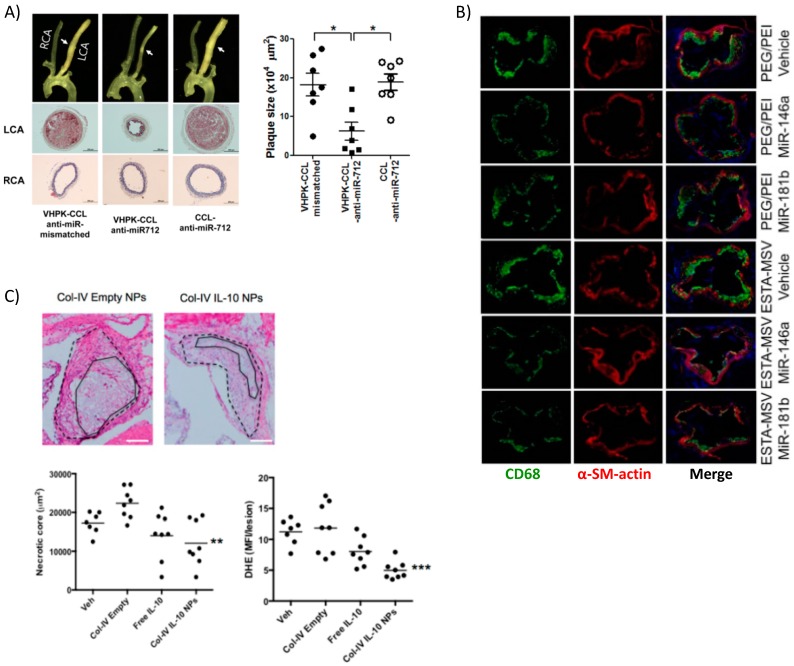
Targeted delivery of biotherapeutics from various nanoparticle formulations. (**A**) Surgical models of atherosclerosis lead to disturbed blood flow in ligated artery (LCA) vs. non-surgery control normal flow (RCA) resulting in overexpression of VCAM-1 used by targeted lipoparticles delivering miRNA. Targeted liposomes (VHPK-CCL anti-miR-712) resulted in decreased plaque size, collagen content, and other markers of inflammation (* *p* < 0.05). Reprinted (adapted) with permission from [51]; (**B**) PEI-mediated delivery of athero-protective miRNA encapsulated in silica microparticles targeting E-selectin reduced overall macrophage content within plaques, marking a reduced inflammatory response. Reprinted (adapted) from [60]; (**C**) Collagen-IV targeted PLGA NPs encapsulating anti-inflammatory cytokine IL-10 also localized to the plaque and reduced necrotic core size and generation of ROS. Reprinted (adapted) with permission from Kamaly, N. et al. Targeted Interleukin-10 Nanotherapeutics Developed with a Microfluidic Chip Enhance Resolution of Inflammation in Advanced Atherosclerosis. ACS Nano 2016, 10, 5280–5292. Copyright 2016 American Chemical Society.

Kheirolomoom et al. devised a liposome formulation encapsulating anti-micro RNA 712 (miR-712) for targeted delivery to the plaques of ApoE^−/−^ mice. The encapsulation allowed for potent in vivo downregulation of miR-712, whose main target is matrix metalloproteinase (MMP) activity, at a dose 80% lower than if given freely without lipid NP encapsulation [51]. MMPs are enzymes produced by immune and other cells to degrade extracellular matrix (ECM) components. Typically, after an inflammatory reaction has occurred, the pro-healing remodeling response requires MMPs [113]. However, in the case of atherosclerosis, a thick and stable fibrous cap over the plaque prevents rupture and subsequent thrombosis, so MMP activity is associated with vulnerable plaques. To target the plaque, the authors made use of the validated VHPK peptide [114], which targets endothelial VCAM-1 and helps to increase not only binding of the NPs to endothelial cells but also their internalization. Nanoparticle-mediated delivery of anti-miR-712 greatly reduced atheroma formation associated with a stable collagen cap (Figure 5A).

In another interesting work, miRNA-146a and -181b were packaged into PEG-PEI NPs that were then loaded into a multistage silicon microporous vessel conjugated with E-selectin targeting aptamers [60]. These specific miRNAs are downregulated in the atherosclerotic inflammatory condition, prompting their selection as therapeutics. The miRNA delivery in male ApoE^−/−^ mice led to a decrease in the expression of chemokines CCL2, 5, 8, and 9 as well as CXCL9. These are well known downstream products of the inflammatory NF-κB signaling pathway, which these miRNAs are reported to inhibit. The authors reported decreased monocyte adhesion to the endothelium and fewer pro-inflammatory immune cells populating and perpetuating inflammation at the plaque site as evidenced by reduced expression of monocyte lineage marker CD68 (Figure 5B). Hence, miRNAs are attractive options for therapeutics and new information is continuously bringing to light their role in atherosclerosis [115].

Short interfering RNA (siRNA) is another type of RNA interference strategy used to regulate protein expression that shows great promise in translation to the clinic. Using a lipid-like nanoparticle formulated from low molecular weight PEI, PEG, and epoxide-terminated hydrophobic polymers [116], Dahlman et al. targeted the endothelium for the delivery of siRNA that downregulated the endothelial cell receptors VE-cadherin and ICAM-2 [67]. Though not specifically targeted to endothelial biomarkers, one hydrophobic polymer nanoparticle formulation was able to accumulate preferentially in the endothelium of C57BL/6 mice. This was reportedly one of many formulations tested in an initial high-throughput in vitro screen in HeLa, murine endothelioma, and pooled human dermal microvascular endothelial cells for RNA knockdown efficiency. The formulation derived from a copolymerization of PEI_600_ and an epoxide-functionalized C_13_ chain. Epoxide ring opening links the hydrophobic chain to PEI amine groups, thus it is not necessarily a co-polymer but rather a long chain cationic lipid. This is confirmed by the multilamellar vesicle (MLV) arrangement of lipids, PEG, and siRNA. The authors reported high efficiency of protein knockdown with the nanoparticle vehicle allowing for measurable therapeutic interventions with as little siRNA as 2 nM. The siRNA decreased infiltration of immune cells into the plaques. One thing to note was this formulation was used in the healthy endothelium of mice, which is significantly different than the damaged and activated endothelial cells lining plaques. It will be interesting to see if this success translates to the athero-prone endothelium.

Using a similar nanoparticle formulation, but focusing more on specific targeting, Chung et al. used in vivo phage display technology to identify peptides that bind activated endothelial cells under oscillatory, or disturbed, flow conditions [59]. Using a partial carotid artery ligation surgery in male C57BL/6 mice, the authors identified peptides that localize more specifically to the ligated artery experiencing oscillatory flow than the non-ligated control. This ensured that the targeting peptides purified through multiple phage pannings of the ligated artery would target only endothelium experiencing disturbed flow. By conjugating these peptides to a PEG-*g*-PEI copolymer nanoparticle encapsulating siRNA against ICAM-1, they reduced ICAM-1 mRNA expression by a third in vivo. As with many nanoparticle-based strategies, there was accumulation in the spleen and kidney. The authors have also shown a likely target of the discovered peptides to be non-muscle myosin heavy chain IIA (NMHCIIA), which is upregulated in the case of disturbed flow. The protein is known for its binding to and regulation of actin in cell migration and adhesion [117] and thus has implications for the cytoskeletal organization of ECs. Morphological changes in endothelial cells arise from their adaptation and remodeling in response to mechanical stimuli such as cyclic stretch and shear stress within the blood vessels [118,119].

#### 3.1.2. Plasmid DNA (pDNA) Delivery

DNA-based gene therapy is one of the few newcomers to atherosclerosis research and has the potential to increase the expression of proteins involved in anti-inflammatory, lipid processing, or other pathways involved in plaque progression. pDNA offers many advantages to delivering proteins. DNA plasmids are easier and cheaper to work with than proteins and if delivered properly can ensure that the protein is produced endogenously where it often needs to be transiently expressed at low doses (pg/mL). DNA delivery held enormous promise at its inception but has faced significant challenges moving past experimental treatments in animal models. There are unique challenges to the delivery of DNA, mainly its need to cross both the cellular and nuclear membrane. Of course, viruses can be counted on to properly perform this task with high efficiency, but there is still apprehension limiting their widespread use including a low cargo carrying capacity, safety concerns, and the possibility for mutation and oncogenesis. Two recent investigations into viral mediated gene therapy in vivo were successful after pre-suppressing the immune system to the virus [120,121], a strategy that could hamper translation of this therapy. However, viruses have been useful to identify potential therapeutic proteins involved in atherosclerosis [122]. For these reasons, researchers have been turning to synthetic and natural polymer-derived nanoparticles for gene delivery [123] and applying insight gained from siRNA delivery to DNA [124].

In an older but interesting strategy immunizing male New Zealand white rabbits on a high cholesterol diet against atherosclerosis, Yuan et al. used chitosan nanoparticles to deliver DNA encoding cholesterylester transfer protein (CETP), which is responsible for transferring lipids between lipoproteins [44]. Chitosan is a natural cationic polysaccharide derived from chitin, present in shellfish, with primary amine groups. It is biodegradable, biocompatible, and even has purported anti-inflammatory properties [125], making it a desirable candidate for biotherapeutic delivery [126]. The authors took advantage of the cationic and mucoadhesive properties of chitosan to craft a nanoparticle vaccine to be delivered intranasally. Vaccinated rabbits mounted an effective immune response against CETP and helped lower its activity for 21 weeks, reducing plaque formation. This initial promising study highlighted the potential of DNA delivery for atherosclerosis, but there is much work to be done with non-viral pDNA gene therapy before reaching the success observed with RNAi-based interventions.

More in vitro characterization of the transfection capabilities and cytotoxicity of cationic polymers must be performed in cells relevant for atherosclerosis. Self-assembled PEI end-capped amphiphilic copolymers made of lactic acid and 2,5-morpholinedione were capable of condensing and delivering DNA to HUVECs [71]. The authors reported little to no cytotoxicity, a common concern with polymer materials, and a biodegradation rate better than that of polylactic acid, a prototypical cationic biopolymer. They claimed the depsipeptide (amide and ester bond donors) bonds within the polymer helped to prolong release of pDNA and contributed to its overall biocompatibility. As mentioned previously, the inclusion of ester bonds in cationic polymers seems to be a key feature allowing for DNA plasmid delivery. Besides the intelligent design of biodegradable gene delivery polymers, a high expression and non-immunogenic plasmid construct known as minicircle DNA (mcDNA) has outperformed traditional therapeutic pDNA in non-viral carriers [127,128], implying that low transgene expression is not as much a concern anymore for non-viral methods.

#### 3.1.3. Protein Delivery

Until the potential of pDNA gene therapy is fully realized, studies delivering the protein itself can offer unique insights into the bioactivity and dosing regimens required for robust in vivo responses. Some early evidence of success comes from Muro et al. who delivered acid sphingomyelinase (ASM) as a treatment for lysosomal storage disease. Though not specifically for atherosclerosis processes, they used fluorescently labeled polystyrene beads with a diameter of 200–300 nm coated with recombinant ASM and an ICAM-1 antibody. The NPs specifically bound to and were taken up by two types of ICAM-1 expressing cells, HUVECs and fibroblasts from patients with Niemann-Pick disease (NPD), and reduced the uptake of lipids [61].

Recently, more advanced and fine-tunable nanoparticles have been devised for the controlled release of targeted protein therapeutics at the site of the plaque. Kamaly et al. utilized a three-channel microfluidic device to fabricate PLGA-PLA copolymer nanoparticles coated with PEG and a collagen IV-binding peptide for targeting [39]. These NPs were able to encapsulate interleukin-10 (IL-10), the potent anti-inflammatory cytokine, for therapeutic delivery to the plaque. The biodegradable NPs were capable of reducing plaque development, necrotic core size, and lowering reactive oxygen species (ROS) in an atherosclerosis mouse model (ldlr^−/−^ on a high fat diet) (Figure 5C). This study adds to the research underlining IL-10 as important in curbing the immune response [129] particularly in atherosclerosis [122,130]. The same collagen-binding peptide was used to target NPs delivering an inflammation resolving peptide mimic of the protein Annexin A1 [38] to ldlr^−/−^ mice on a high fat diet. Targeted delivery of the peptide, known as Ac2–26, increased collagen deposition and established a thick fibrous cap, among other markers indicating a stable plaque. These studies highlight the high therapeutic potential of targeted anti-inflammatory or pro-healing nanoparticle therapeutics in resolving inflammation, a key step absent in atherosclerosis [131]. Being that there are few viable options for anti-inflammatory therapy in clinical trials thus far, the most promising is a non-targeted antibody against the pro-inflammatory IL-1β [132], there is room for targeted nanotherapies to address non-resolving inflammation in atherosclerosis in the future.

#### 3.1.4. Cell Delivery

Alternatively, Adamo et al. investigated the delivery of cells as therapeutics. By introducing biodegradable PLA-based magnetic nanoparticles in vitro into rat aortic endothelial cells, the authors were able to load ECs with nanomagnets [75]. The treatment was intended for mechanical injury following stent implantation that can often leave the endothelium damaged but this work could be applicable for atherosclerosis. The complex shear stress profile around plaques coupled with the body’s immune response has complicated targeting plaques [133]. Fifteen minutes of magnetic field was capable of holding the cells in place to withstand the forces of blood flow, allowing the cells to attach and repopulate the damaged area in male Lewis rats. This early study shows potential in targeting cells themselves to the plaques using nanoparticles.

### 3.2. Delivery of Drugs

Nanoparticle encapsulation of drugs has many benefits including protecting molecules from degradation, solubilizing hydrophobic or pH-sensitive drugs, ensuring molecules are locally concentrated where they are needed, reducing off-target effects, and lowering the overall dose required for activity. These possibilities greatly increase the potential for small molecule treatments in atherosclerosis, especially at a time when an estimated 70% of cardiovascular clinical events cannot be prevented with available drugs [134]. Thus far, the majority of small molecule interventions in atherosclerosis have been limited to lipid-lowering statins, the clinical gold standard for treatment. However, through the unique properties conferred by nanoparticles, a wider range of molecules are becoming suitable candidates such as anti-inflammatory steroids [49,135]. One advantage driving these strategies forward is the increased fine-tunability of therapeutic delivery enabled by nanoparticles and their unique material properties. These are typically lipid or polymer-based formulations that do not require inorganic regions such as in most NPs used for imaging. Natural and synthetic polymers can be designer-functionalized through a wide range of chemistries to produce the ideal formulations capable of immune cell evasion (an increasing standard for in vivo studies), targeting, and even more complex stimuli-responsive results (pH, shear stress, etc.) as discussed earlier. A brief discussion of exciting breakthroughs in NP-mediated drug delivery will follow but more extensive reviews dedicated to this topic can be found [24,112].

One prominent example of NP-mediated drug delivery is for methotrexate, which is typically used as a chemotherapeutic for cancer at higher doses but is widely used as a suppressor of inflammation at lower doses (10–25 mg/week) [136]. This naturally lends itself to nanoparticle-encapsulated delivery. Bulgarelli et al. packaged methotrexate within a lipid nanoemulsion resembling low density lipoprotein (LDL) in New Zealand rabbits. The rabbits were fed a high cholesterol diet for 60 days and received NPs for the last 30 days [47]. The authors reported a decrease in the number of characteristic pro-inflammatory proteins (TNF-α, MCP-1, MMP-9, etc.) within the intimal blood vessel layer along with an upregulation of IL-10, further distinguishing this approach as an anti-inflammatory treatment. The NP-encapsulated methotrexate treatment was as effective as that given in a commercial solution but can potentially avoid toxic side effects sometimes seen with low-dose methotrexate [136].

**Figure 6:**
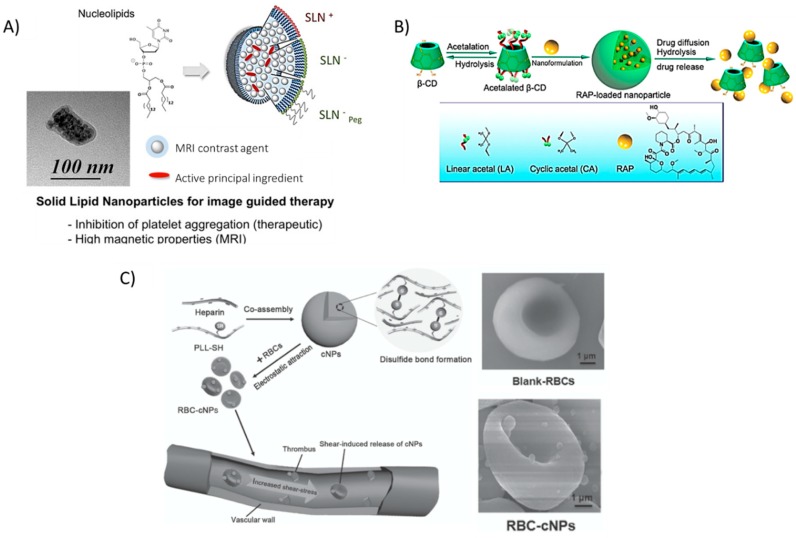
Recent nanoparticle formulations enhancing drug delivery properties in atherosclerosis. (**A**) Solid lipid nanoparticles (SLN) are typically formed by lipids in various phases, however, the novel use of nucleolipids allows for added fine-tunability and stability, leading to the ability to encapsulate active principal ingredients (API) such as the platelet inhibitor prostacyclin (PGI2) and imaging modalities (MRI contrast agents). Reprinted (adapted) with permission from Oumzil, K. et al. Solid Lipid Nanoparticles for Image-Guided Therapy of Atherosclerosis. Bioconjug Chem 2016, 27, 569–575. Copyright 2016 American Chemical Society. (**B**) hydrophobic pockets within β-cyclodextrin (BCD) molecules allow for the encapsulation of potent drugs, such as rapamycin (RAP). The low toxicity and wide range of functionalization possibilities make BCD a promising tool for many studies. Reproduced (adapted) with permission from [56]. (**C**) Disulfide-linked Poly(l-lysine) (PLL) and heparin, a well-known natural anti-coagulant, form cationic nanoparticles that can adhere to red blood cells and ‘hitch a ride’ to the site of thrombus formation, releasing heparin as the particles degrade. Reproduced (adapted) with permission from [55].

Similarly, encapsulating prostacycline (PGI2), an inhibitor of platelet aggregation, within novel nucleoside-liposomes allowed Oumzil et al. to lower the dosage of this powerful molecule [50]. The authors encapsulated both PGI2 and an iron oxide MRI contrast agent for simultaneous therapy and in vivo tracking within solid lipid nanoparticles (SLN), making this a hybrid organic/inorganic theranostic formulation (Figure 6A). Such solid lipid nanoparticles showed successful inhibition of clotting in human blood from healthy volunteers and better magnetic relaxivity properties as compared to commercial MRI contrast agent Feridex^®^, a superparamagnetic iron oxide (SPIO) colloidal formulation [137]. The in vitro/ex vivo NP therapy inhibited platelet aggregation when platelet-rich plasma (PRP) was incubated with clotting agonists adenosine 5′-diphosphate (ADP) and thrombin receptor-activating peptide-6 (TRAP-6).

Dou et al. also encapsulated a potent anti-inflammatory drug typically used in post-transplantation therapy in a novel acetalated β-cyclodextrin formulation [56]. By varying the degree of acetalation, the authors exhibited control over the release of rapamycin (Figure 6B). These rapamycin-releasing NPs led to a significant decrease in aortic lesions in atherosclerotic mice. The authors also noted other typical markers of atherosclerosis that were reduced (MMP-9, macrophage content, plaque area, and necrotic core) while markers of plaque stability were increased (smooth muscle cell infiltration, collagen deposition, and relative lumen area).

Red blood cells (RBCs), an integral component of blood responsible for the transportation of oxygen to all tissues, could be an important delivery tool to shuttle therapeutics to the site of growing plaque. Chen et al. absorbed nanoparticles, formulated between heparin and thiol-functionalized PLL [55], onto red blood cells isolated from female BALB/c mice. The added thiol groups enabled spontaneous cross-linking via the formation of disulfide bonds without the need for other reactants. Instead of incorporating PEG, the authors reasoned that ‘hitching a ride’ on long-circulating RBCs could impart on the absorbed particles a significantly increased bioavailability. Furthermore, at the elevated shear stress level of 10 Pa typical around stenosed vessels, the authors noted an increased release of the nanoparticles from the cell surface as compared to a pressure of 1 Pa, typical in non-occluded vessels. Biodegradation of the polymers resulted in free heparin available, a well-known anti-coagulant. Furthermore, macrophage uptake of RBC-absorbed NPs was reduced in vitro, suggesting that incorporating NPs onto cells could possibly help to evade recognition by the immune system (Figure 6C); especially as concerns begin to rise about the immunogenicity of PEG coatings [138].

Thus, as we learn more about the molecular biology of atherosclerosis, we can ‘smart design’ NP properties based on their materials to exploit certain stimuli in the disease of interest, such as the relationships between oscillatory flow, increased plaque formation, and uptake of shear stress responsive nanoparticles.

## 4. Other Promising Approaches for Atherosclerosis Using Nanoparticles

### 4.1. Nanoparticles as Sensors and Detectors of Atherosclerosis Progression

The detection of vulnerable plaques has become a sought-after goal for quite some time in atherosclerosis research, especially using noninvasive or radiation-free techniques. Nanoparticles have been used for sensing and/or detecting various aspects of atherosclerosis progression and as above, many of these responses are direct results of the material chosen to fabricate the nanoparticle. The simplest process to quantify seems to be the uptake of lipids by professional phagocytes. This is the ongoing process associated with plaque growth and necrotic core enlargement. Ankri et al. used gold nanorods to visualize phagocytes [139]. Gold nanorod uptake by macrophages, derived from human monocytes, increases the absorption coefficient of the tissue and decreases the amount of reflected light. Thus, the signal from diffusion reflectance (DR) imaging between healthy and balloon-injured rat arteries was significant. The increased uptake of nanorods within the injured artery was directly verified by ex vivo imaging via computer tomography. A similar method was used by de Oliveira et al., only substituting the pure gold nanoparticles for aminolevulinic acid-functionalized gold NPs. This agent becomes fluorescent through a biosynthetic pathway, providing an increased level for detection [140].

Fluorescence molecular tomography (FMT) represents an alternative technology to detect plaques at risk of rupture. Nahrendorf et al. coated polymer nanoparticles with enzyme-digestible probes that became fluorescent after enzymatic activity [141]. The authors were able to pinpoint vulnerable plaques in ApoE^−/−^ mice that showed increased activity of MMPs in a whole mouse imaged non-invasively. To increase resolution, however, they included CT imaging capabilities as well. This study could be useful in providing a framework for the non-invasive imaging and quantification of vulnerable plaques as well as in monitoring the success of a treatment, as the authors show can be done after administering atorvastatin. These exciting results raise the potential for smart-designed nanoparticles in the simultaneous diagnosis and tracking of treatments in atherosclerosis.

### 4.2. Nanoparticle-Assisted Modelling of Atherosclerosis Progression

The advancements made in imaging, sensing, and detection of plaques and the blood flow patterns contributing to atherosclerosis have also allowed for gains to be made in the field of modelling. Gitsioudis et al. combined high resolution MRI imaging with computed tomography angiography (CTA) to image complex flow patterns in the vessels and differentiate between low and high shear stresses in thoracic arteries of hyperlipidemic rabbits [76]. The dynamic data they received from these imaging methods was used to create 3D models of fluid flow within the arteries, which the authors then used to predict atherosclerosis progression and general vascular inflammation based on low endothelial shear stresses. Models could be used in conjunction with other methods mentioned above to track plaque development over time [142]. Especially if done non-invasively, data could be generated for each patient so that truly personalized medicine approaches can be investigated.

Modelling can also uncover the physics governing nanoparticle uptake and this information is directly applicable to future strategies. For example, the size of nanoparticles, which varies considerably depending upon the material (tens of nm for inorganic materials to hundreds of nm or even microns for polymers), can significantly influence NP dynamics within the vessels [143] as well as NP uptake by cells once particles arrive at the target site. Gonzalez-Rodriguez et al. probed these interactions in a computational model developed to show how nanoparticles coated with an ICAM-1 antibody would be taken up by endothelial cells [80]. ICAM-1 is activated by inflammation in the plaque and then differentially expressed by endothelial cells lining the plaque (20- to 100-fold increase over quiescent endothelial cells). Thus, it is a good marker and target for the inflamed endothelium and indeed appears often in Table 2.

Internalization time for NPs ranged between 2 and 3 s for NPs with diameters of 50 to roughly 220 nm according to the authors. The time increases for smaller NPs as they are probably taken up by a different mechanism than CAM-mediated endocytosis. Similarly, particles larger than 220 nm saw increased internalization times due to the difficulty of cell membrane deformation and the physical accommodations that the cell had to make to take in large particles. However, the model predicts that NPs up to around 1 μm may enter endothelial cells through some type of membrane wrapping [80]. The internalization time is also dependent on bond formation time between the NP ligand and cell receptor, which could also direct investigators to vary surface ligand density as a strategy for increased uptake as mentioned in previous sections. This study explores some fundamental boundary conditions that must be met for successful NP-mediated therapeutic targeting.

The integration of these modelling and sensor approaches could culminate in one of the latest trends in in vitro cell culture technology; recapitulating complex cell-cell and cell-material interactions using microfluidics. The so-called disease-on-a-chip model may still be far off for atherosclerosis, but complex processes especially those involving flow and shear stress, can be successfully modelled. Zheng et al. developed a culture platform for endothelial cells wherein they could vary both fluid shear stress and cyclic stretch, as is often experienced by ECs stimulated by underlying vascular muscle tissue, to have a more physiological or pathological environment using a microfluidic chip [118]. The device gives vascular endothelial cells a much-needed upgrade in in vitro culture conditions that more resemble their natural in vivo environment. The potential applications begin with probing the endothelium for more physiologically relevant answers and extends right through to evaluating new drugs and experimental nanomedicines. The authors report that platinum nanoparticles (PtNPs), which are capable of reducing ROS, showed similar effects on their chip as in vivo. These PtNPs have shown promising experimental results, yet their clinical safety and applicability remains unknown, making them an ideal candidate for chip-based experiments.

## 5. Conclusions and Perspectives for Future Work

**Figure 7:**
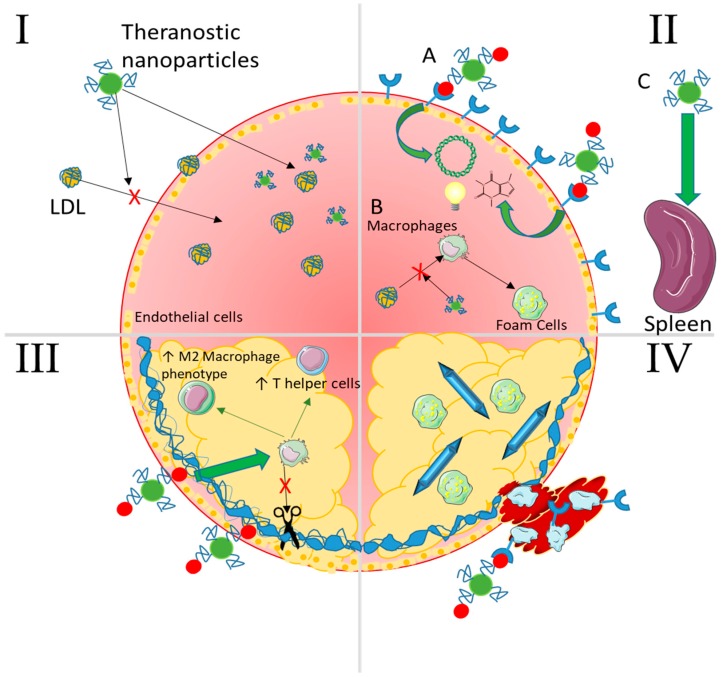
Summary of selected nanoparticle strategies capable of intervening at any stage of atherosclerosis. I—Lipid Accumulation NPs passively accumulate as well as lipids because of their size and surface functional chemistries and can even be made to mimic LDL. In addition, they can carry imaging contrast agents, drugs to lower cholesterol levels, and even nucleic acids to genetically regulate expression of cholesterol ester transfer proteins (CETPs). II—Non-resolving inflammation (**A**) NPs can target inflammatory recruitment receptors with conjugated moieties (peptides, aptamers, antibodies) and enter the plaque to deliver therapeutic nucleic acids, drugs, and/or imaging agents. (**B**) They can also interfere with macrophage uptake of oxLDL to form foam cells via competitive interaction with scavenger receptors. (**C**) Passive accumulation in the spleen allows for therapeutic delivery to the progenitor cells and macrophages that would normally egress to populate the plaque. III—Plaque cap destabilization NPs targeting damaged and exposed components of the fibrous cap (collagen/elastin) can deliver therapeutics and/or imaging agents as in panel II. These can lower destructive activity of cytokines and enzymes secreted by macrophages and help to stabilize the cap by inducing an anti-inflammatory environment (T regulatory and helper cells and M2 macrophages) through localized immunomodulation or pro-healing enzymes such as tissue inhibitor of metalloproteinases (TIMPs). IV—Thrombosis NPs can target receptors expressed on platelets as well as on activated endothelial cells such as P-selectin. These can deliver anti-thrombogenic drugs locally or be used to image thrombus formation via contrast agents. Advanced cholesterol crystals can also be dissolved in an attempt to decrease the dangerous lipids resident in the plaque.

Many types of nanoparticles have made their way into all areas of research into atherosclerosis including its pathology, sensing, therapeutic delivery strategies, imaging, and development of more physiologically relevant models. From a bioengineering perspective, the convergence of advancements in both atherosclerosis pathology and nanoparticle materials research provides a unique nano-sized window through which we can see the future of diagnosis and treatment. In fact, the gamut of advanced nanoparticle formulations available today is so wide that researchers seem to be able to therapeutically intervene in atherosclerosis at every stage of disease progression (Figure 7). With the advances in materials that allow for fine tuning of NP properties, tracking, reporting, and sensing, it will not be long before nanoparticles become standard tools to uncover the underlying events in atherosclerosis progression. At the same time, researchers have found and continue to find new and interesting therapeutics to encapsulate within NPs for delivery, each with its own unique set of design criteria and constraints. Furthermore, NPs can be evaluated on smaller and smaller microfluidic models as we approach true disease-on-a-chip models.

The delivery of biotherapeutics seems uniquely suited to addressing the underlying inflammation at the heart of atherosclerosis. The aforementioned strategies involving the delivery of RNAi, which led to an increased deposition of collagen and stable fibrous cap, were promising. Furthermore, the delivery of biotherapeutics has the potential to extend into the exciting areas of gene editing, whole genome, and personalized medicine strategies in the context of atherosclerosis. However, there are still relatively few studies and more work needs to be done to corroborate these findings in more and larger animal models.

The translational potential of NP-mediated strategies remains unknown as regulatory agencies must adapt novel methods of evaluation for these complex technologies. What seems necessary for new strategies to succeed is interdisciplinary cooperation between research fields. It is no longer enough to design and synthesize novel NPs and evaluate their efficacy/safety in one cell type in a petri dish. They must be tested in vivo or in as close to an in vivo environment as possible on microchips or in silico. The importance of screening NP materials for toxicity during experiments in vitro and in vivo should not be overlooked as well. Targeting the endothelium in a safe and effective way is the ultimate goal for NP-mediated therapies [133], but it may induce added damage, which can accelerate atherosclerosis lesion formation [144].

There is no shortage of constraints in using nanotechnology for any purpose in biology. However, researchers continue to identify novel targets for imaging, diagnosing, and treating atherosclerosis using nanoparticles. With the recent combination of therapeutics delivered directly and specifically to the vulnerable plaque, a targeted NP-based theranostic treatment based on NP-elucidated pathologies is becoming more possible.

## Figures and Tables

**Table 1:** Summary of common materials used to fabricate NPs used in atherosclerosis research studies selected for this review.

Common NP Materials	Drug Delivery	Cell/Gene/Protein Delivery	Imaging
PLA/PLGA	[36,37]	[38,39,40,41]	[42]
Chitosan	[43]	[44]	[45]
Hyaluronic Acid	-	-	[46]
Liposomal Lipids ^a^	[47,48,49,50]	[51]	[52,53,54]
PLL	[55]	-	-
Cyclodextrin	[56,57]	-	-
PAA	[43]	-	-
PEG ^b^	[58]	[38,39,59,60]	-
Sebacic Acid	[58]	-	-
Polystyrene	-	[61]	[62,63]
α-Elastin	[37]	-	-
Polypyrrole	-	-	[46]
Gold	-	[64]	[65,66]
Synthetic Polymer 7C1	-	[67,68,69]	-
Perfluorocarbon	-	-	[54,70]
PEI	-	[59,60,71]	-
Silicon	-	[60]	-
Gadolinium	-	[72]	[52,53,73,74]
Iron Oxide	-	[75]	[76,77,78]

^a^ Typical liposomal lipids include cholesterol, 1,2-Distearoyl-sn-glycero-3-phosphoethanolamine (DSPE), *N*-[1-(2,3-Dioleoyloxy)propyl]-*N*,*N*,*N*-trimethylammonium (DOTAP), phosphotidylcholine, and those similar. ^b^ PEG in this table includes incorporation as a main functional component (i.e., co-polymer) rather than surface coating.

**Table 2:** A non-exhaustive list of targets recently identified through various NP-mediated targeting of atherosclerosis, mainly for imaging purposes.

Process	Target	Targeting Moiety	Vehicle	In Vivo Study	Reference
**Apoptosis**	Phosphatidyl serine	Annexin V	SPION	Hyperlipidemic Rabbit	[84]
			USPIO	ApoE^−/−^ mice	[85]
		Peptide R826	USPIO	ApoE^−/−^ mice	[86]
	Membrane Potential (ΔΨ_m_)	Triphenyl phosphonium (TPP) cation	HDL-inspired polymer-lipid hybrid NP	Rat	[87]
**Angiogenesis**	α_V_β_3_	Peptidomimetic antagonist	PFC-lipid NPs	Hyperlipidemic Rabbit	[88]
**Calcification**	Ca^2+^	Succinate derivatives	IONP	ApoE^−/−^ mice	[89]
		Citrate coating	VSOP	Hyperlipidemic Rabbit	[90]
**Leukocyte infiltration**	C-C chemokine receptors	^64^Cu-labelled vMIP-II	PMMA/PEG core-shell NPs	ApoE^−/−^ mice	[91]
**Macrophage activity**	CD44 or Stablin-2	Hyaluronic Acid (HA)	Hydrophobically modified HA NPs	ApoE^−/−^ mice	[92]
	CD36	Specific oxidized phospholipids	Gd-entrapped carbon cage within liposome	ApoE^−/−^ mice	[93]
	p32	Lyp-1 peptide	HSP-1 self-assembled cage	Arterial ligation surgery in mice	[94]
	Scavenger receptor SRA-1	Inherent ability of polymers	PEGylated aliphatic mucic acid derivatives	Rats	[95]
	Mannose receptor	Mannose	Hydrophobically modified glycol chitosan NPs	ApoE^−/−^ mice	[96]
	Unknown	Inherent ability of protein cage	Human recombinant protein cage	FVB mice	[97]
**Fibrous cap formation**	Collagen	EP-3533 peptide	PEGylated HDL-like NPs	Reversa mice	[98]
**Elastic lamina damage**	Elastin	Antibody	PLA NPs	ApoE^−/−^ mice	[42]
**Endothelial inflammation**	E-selectin/VCAM-1	Antibodies	Commercial polystyrene NPs	ApoE^−/−^ mice	[62]
	P-Selectin/VCAM-1	Synthetic polymer targeting P-selectin (PAA-sLe^x^) and VCAM-1 antibody	PFC-filled lipid microbubbles	NA	[22]
	P-selectin	Antibody	PEGylated dextran/IONP	ApoE^−/−^ mice	[99]
	VCAM-1	Nano antibody fragment	^18^F-labelled antibody	ApoE^−/−^ mice	[100]
		Peptide R832	USPIO	ApoE^−/−^ mice	[86]
		Peptide VHPKQHR	PFC core w/lipid surfactant	ApoE^−/−^ mice	[70]
		Antibody	PFC-filled ultrasound microbubbles	NA	[101]
**General inflammation**	IL-4 receptor	IL-4 analogous peptide	Hydrophobically modified glycol chitosan NPs	Ldlr^−/−^	[45]
	MRP8/14 (calprotectin)	Antibody	Gadolinium-loaded liposomes	ApoE^−/−^ mice	[102]
**Thrombosis**	Platelets	RGD Peptide	IONP-loaded PLGA-chitosan core-shell NPs	Sprague-Dawley rats	[103]
	Thrombin	PPACK (Thrombin inhibitor)	PFC core with phospholipid surfactant	ApoE^−/−^ mice	[104]

**Table 3:** Overview of highlights in NP-mediated targeted delivery of biotherapeutics recently used to attenuate atherosclerosis.

Biotherapeutic Delivered (Target)	NP Targeting Moiety	NP Material	Result	Reference
siRNA (ICAM-1)	in vivo phage display-identified peptide targeting NMHC IIA	B-PEI crosslinked with added disulfide bonds and conjugated to peptide via heterobifunctional PEG	NPs target athero-prone regions and lead to ICAM-1 knock-down	[59]
siRNA (ICAM-2)	None, but preferentially accumulates in pulmonary ECs	Hydrophobically modified (C_13_) PEI formed into liposome-like NPs with PEG incorporated	Significant in vitro and in vivo mRNA silencing in endothelial cells for a variety of vascular pathologies, particularly Lewis Lung Carcinoma (LLC).	[67]
Ac2-26 peptide from Annexin A1 (N-formyl peptide receptor FPR2/ALX)	Collagen IV-binding peptide	PLGA bioconjugated to PEG and peptide	NPs target athero-prone regions and reduce lesion size, oxidative stress, increase collagen, and enhance athero-protective effects.	[38]
Interleukin-10 (IL-10 receptor)	Collagen IV-binding peptide	PLGA-PLA copolymer with PEG coating and bioconjugated peptide	NPs target athero-prone regions and reduce lesion size, cap thickness, neutrophil infiltration, and immune cell responses to inflammatory stimuli.	[39]
Anti-miRNA (miR-712, known association with MMPs)	VCAM-1 targeting peptide	Liposomes formed from cationic lipids, PEG-lipids, and peptide-PEG-lipids	Specific targeting of ECs in vivo under oscillatory/low shear stress leading to decreased plaque size, increased cap size, and decreased destructive enzymatic activity.	[51]
MiRNA (miR-146a and miR-181b)	E-selectin targeting peptide	PEG-g-PEI:miRNA NPs encapsulated within silicon microparticles	Improved relaxation of vascular endothelium ex vivo, reduced chemotactic ligand expression/monocyte adhesion in addition to lesion/collagen area and macrophage, while increasing SMC migration.	[60]
